# Challenging the concept that eumelanin is the polymorphic brown banded pigment in *Cepaea nemoralis*

**DOI:** 10.1038/s41598-020-59185-y

**Published:** 2020-02-12

**Authors:** Susanne Affenzeller, Klaus Wolkenstein, Holm Frauendorf, Daniel J. Jackson

**Affiliations:** 1 Department of Geobiology, Georg-August University of Göttingen, Goldschmidtstrasse 3, 37077 Göttingen, Germany; 2 Institute of Organic & Biomolecular Chemistry, Georg-August University of Göttingen, Tammannstrasse 2, 37077 Göttingen, Germany

**Keywords:** Molecular evolution, Bioanalytical chemistry

## Abstract

The common grove snail *Cepaea nemoralis* displays a stable pigmentation polymorphism in its shell that has held the attention of scientists for decades. While the details of the molecular mechanisms that generate and maintain this diversity remain elusive, it has long been employed as a model system to address questions related to ecology, population genetics and evolution. In order to contribute to the ongoing efforts to identify the genes that generate this polymorphism we have tested the long-standing assumption that melanin is the pigment that comprises the dark-brown bands. Surprisingly, using a newly established analytical chemical method, we find no evidence that eumelanin is differentially distributed within the shells of *C. nemoralis*. Furthermore, genes known to be responsible for melanin deposition in other metazoans are not differentially expressed within the shell-forming mantle tissue of *C. nemoralis*. These results have implications for the continuing search for the supergene that generates the various pigmentation morphotypes.

## Introduction

Many molluscs adorn their shells with complex and colourful geometric patterns that fulfil a variety of ecological roles. Despite considerable interest in the aesthetic beauty of these pigments (which can also be of significant commercial relevance), little is known about their chemical nature or the molecular mechanisms that generate them. With highly variable and colourful patterns of shell pigmentation the common grove snail, *Cepaea nemoralis*, has long been a textbook example of phenotypic variability and a model system for evolution, ecology, population genetics and even global change^1–5^. Early studies established that the shell background colour, the number of dark pigmented bands on the shell and several other shell-pigment characteristics are inherited in a Mendelian fashion^6,7^, suggesting that the loci for these characters are tightly clustered into what can be referred to as a “supergene”^8–10^. In addition, it has long been assumed that melanin (specifically eumelanin) is the pigment that comprises the dark pigmented bands on shells of *C. nemoralis*^11–13^, and that melanins are common in mollusc shells^11^. If melanin is indeed the pigment that comprises the dark brown bands, this could greatly inform the ongoing search for the supergene that controls this polymorphism. Here we test this assumption using two techniques: a newly developed sensitive analytical chemistry method that we recently reported for melanins^14^; and RT-qPCR against melanin pathway genes with reference genes specifically validated for *C. nemoralis*^15^.

## Results and Discussion

**Figure 1:**
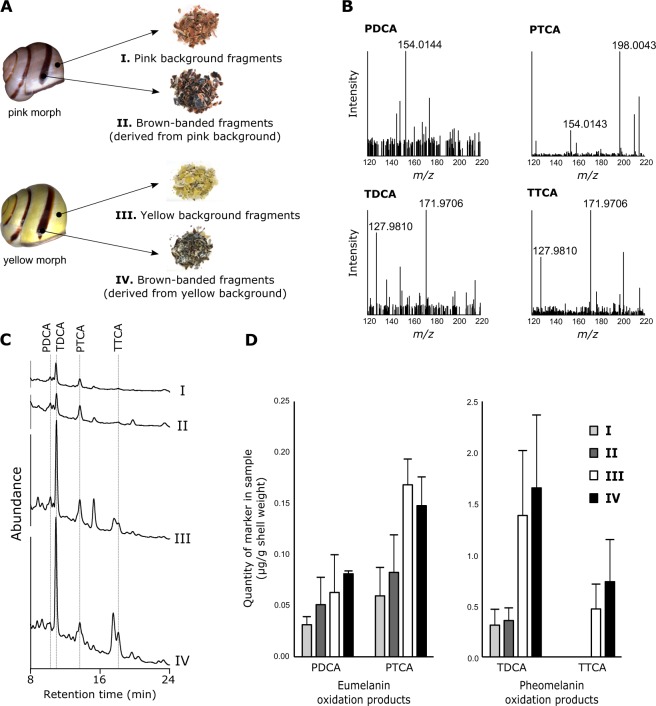
Quantification of eu- and pheomelanin markers from colour-sorted shell fragments of *C. nemoralis*. (**A**) Shell fragments used for LC-UV-MS were sorted by colour. (**B**) Mass spectra for all four melanin oxidation markers as measured in a representative yellow background sample (III from panel A). (**C**) Representative UV chromatograms used for quantitation of melanin oxidation markers. (**D**) Eumelanin and pheomelanin oxidation products were quantitated by HPLC-UV with external calibration. Eumelanin (PDCA and PTCA) and pheomelanin (TDCA and TTCA) oxidation markers were quantified for each shell colour fraction and normalised to initial sample weight. n = 3 for each shell colour fraction with each replicate comprised of 8 shells.

Our initial analytical chemistry experiments on whole shells of the common yellow banded morph of *C. nemoralis* detected all four common melanin oxidation products: pyrrole-2,3-dicarboxylic acid (PDCA) and pyrrole-2,3,5-tricarboxylic acid (PTCA) for eumelanin, and thiazole-4,5-dicarboxylic acid (TDCA) and thiazole-2,4,5-tricarboxylic acid (TTCA) for pheomelanin, albeit for eumelanin at levels significantly lower than the darkly pigmented shells of *Mytillus edulis*^16^. To further investigate the spatial distribution of eu- and pheomelanin in *C. nemoralis* shells we have now conducted quantitative measurements of colour-sorted shell fragments (brown-bands derived from pink background, pink background, brown-bands derived from yellow background, and yellow background; Fig. 1A). All four colour-sorted shell fractions contained the markers PDCA and PTCA (Fig. 1B), indicating a broad distribution of eumelanin, however we could not detect significant differences in the amounts of these eumelanin markers between brown-bands and background shell fractions for either yellow or pink shells (Fig. 1C,D). We also detected the pheomelanin markers TDCA and TTCA in both brown-bands derived from yellow background and yellow background shell fractions in relatively high abundance, but again with no significant difference between the two (Fig. 1D). In pink shells we detected far less TDCA in both fractions while TTCA was below the limit of quantitation but could still be detected (Table 1).

**Table 1:** Melanin oxidation products normalised to shell weight.

Sample	PDCA (µg/g) ± SD	PTCA (µg/g) ± SD	TDCA (µg/g) ± SD	TTCA (µg/g) ± SD
pink morph background	0.03 ± 0.01	0.06 ± 0.03	0.31 ± 0.12	<LOQ
pink morph band	0.05 ± 0.03	0.08 ± 0.04	0.36 ± 0.12	<LOQ
yellow morph background	0.06 ± 0.04	0.167 ± 0.03	1.38 ± 0.64	0.47 ± 0.24
yellow morph band	0.08 ± 0.00	0.145 ± 0.03	1.65 ± 0.71	0.74 ± 0.4

For each sample n = 3, means ± standard deviation. External calibration was set with 9-point calibration curves. Limit of quantitation (LOQ) was set at 10:1 signal-to-noise ratio (LOQ_PDCA_ = 0.08 µg/ml), LOQ_PTCA_ = 0.10 µg/ml, LOQ_TDCA_ = 0.25 µg/ml, LOQ_TTCA_ = 0.33 µg/ml).

**Figure 2:**
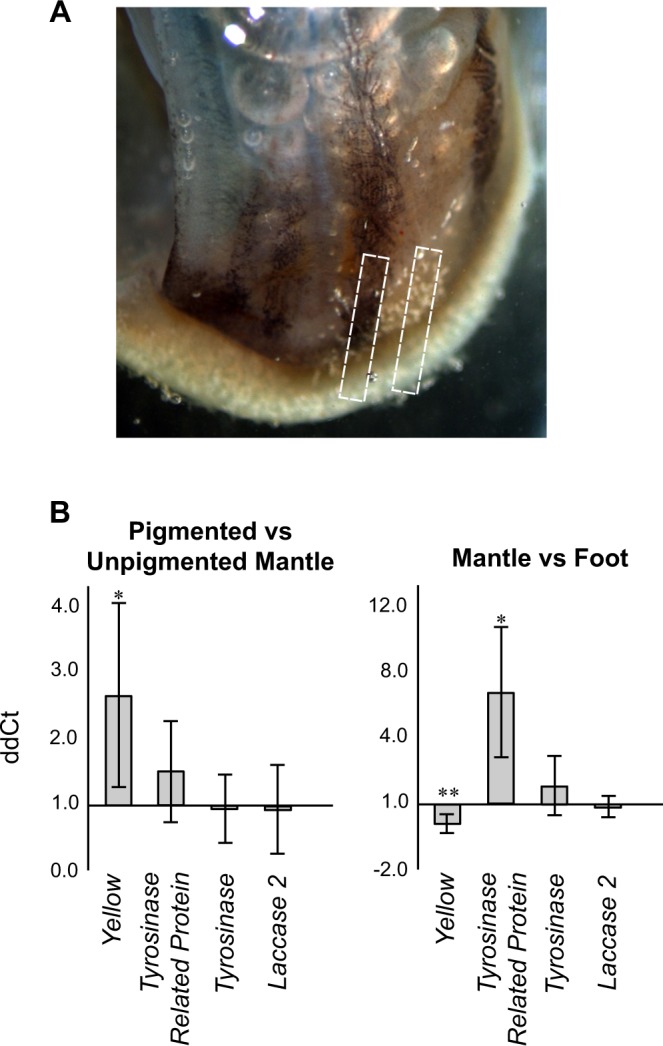
qPCR analysis of normalised relative expression values (ddCt) of four melanin pathway genes. (**A**) RNA was extracted from pigmented and immediately adjacent non-pigmented mantle tissue (white boxed regions). (**B**) Expression levels were compared between dark-brown pigmented mantle tissue vs. non-pigmented mantle tissue, and between whole mantle vs. foot tissue. For each tissue n = 6. * indicates p ≤ 0.05, ** indicates p ≤ 0.01 as per a Mann-Whitney test.

To further investigate the presence of melanin in the brown bands of *C. nemoralis* shells we studied the expression of four known melanin synthesis pathway genes in regions of the mantle responsible for depositing the brown bands in to the shell (Fig. 2A). We quantified gene expression via qPCR using reference genes we recently validated for *C. nemoralis*^15^. We selected the genes *Yellow* and *Laccase 2* (known to be involved in melanin synthesis in insects^17–21^). Furthermore Miyashita and Takagi^22^ recently showed that a tyrosinase is involved in the subtle pigmentation of oyster pearls, while Vicario *et al*.^13^ previously suggested that either a *Tyrosinase* or a *Tyrosinase related protein* is involved in the melanic pigmentation of *C. nemoralis* shells. Phylogenetic analyses of the four genes that we studied are provided in the Supplementary Material (Supplementary Files 1–3).

Despite considerable variation *Cnem-Yellow-like* was significantly differentially expressed in pigmented vs. non-pigmented regions of the mantle (p < 0.05 but not <0.01; Fig. 2B). However *Cnem-Yellow-like* transcripts were significantly more abundant (approximately three fold) in foot tissue relative to mantle tissue overall. This suggests that either *Cnem-Yellow-like* has pleiotropic roles in the foot and mantle, or that there is another rate-limiting enzyme or process that masks the differential activity of *Cnem-Yellow-like* between mantle and foot tissues, or that *Cnem-Yellow-like* is not involved in melanogenesis in *C. nemoralis*. Supporting this latter scenario the *Cnem-Yellow-like* homolog we identified here has relatively low sequence similarity (25% identity) with insect isoforms known to be involved in melanogenesis, and does not phylogenetically group with canonical insect Yellow proteins (Supplementary File S2). *Cnem-Tyrosinase related protein* was significantly upregulated in mantle vs. foot, however comparisons between pigmented and non-pigmented mantle tissue revealed no significant differences in expression (Fig. 2B). In addition, *Cnem-Tyrosinase* and *Cnem-Laccase 2* were equally expressed between foot and mantle tissues and also between pigmented and non-pigmented mantle tissue (Fig. 2). Although these qPCR results in themselves offer no support for the evidence of melanin synthesising activity in the mantle of *C. nemoralis* we must acknowledge that it remains possible that an inhibitory mechanism may differentially operate in pigmented and non-pigmented tissues.

While we do detect the presence of eu- and pheomelanin oxidation markers in shells of *C. nemoralis*, the spatially quantified LC-UV-MS and RT-qPCR data we present here suggests that eumelanin is not the dark banded pigment used by *C. nemoralis*. As far as we are aware the biochemical evidence that suggested melanin to be the responsible pigment in *C. nemoralis* was published more than 80 years ago^12^. Interestingly Comfort later states in regard to the banded pigment in *C. nemoralis* that “Attempts to attack the chemistry of the pigments with melanin inhibitors such as phenyl thiourea have not so far succeeded….” and that “Very little can be said of these colour patterns in terms of chemistry. The black pigment (‘melanocochlin’) is alkali-soluble^12,23^”. Nonetheless, subsequent works refer to pigmented cells in the mantle as melanocytes^24^ and have worked from the assumption that melanin is the brown-banded pigment. For example the upregulation of either *tyrosinase* or *tyrosinase-related* genes in *C. nemoralis* mantle tissue has been interpreted as evidence that melanin synthesis is occurring there^13^. However it is known that these enzymes also function to sclerotize the periostracum and are not necessarily involved in melanin synthesis^25,26^. We previously demonstrated that the brown-banded pigment in *C. nemoralis* is apparently not a chromoprotein^27^, and the results we present here indicate that a non-melanogenic mechanism is operating in the mantle tissue of *C. nemoralis* to deposit a dark-brown pigment in a banded fashion. A recent review on molluscan shell colour by Williams^28^ highlighted the lack of data that unequivocally demonstrates the presence of melanin in molluscan shells, and our own recent survey of molluscan shells with brown/black pigments suggests that in many cases these pigments are not melanin^16^. What then are the brown-banded and background pigments in *C. nemoralis* shells? Currently our LC-UV-MS analyses do not reveal any hints as to the nature of these molecules, however a macromolecular composition may be supposed.

A chemical understanding of what the dark-banded pigment and the various background pigments are, together with all of their respective synthesis pathways and associated genes, would assist efforts in identifying the supergene that is thought to regulate the famous *Cepaea* colour polymorphism. Such a holistic understanding of this polymorphism would provide deep insight into the evolutionary biology of this system, and would allow *C. nemoralis* to join the short list of species for which a polymorphic trait is understood on molecular, phenotypic, population and ecological scales.

## Methods

### *Cepaea nemoralis*

Six living animals and approximately 100 empty shells of *C. nemoralis* were collected at University of Göttingen, Germany (51°33′24.0″N 9°57′27.3″E). Empty shells were cleaned and dried, then crushed. Shell pieces were sorted according to replicate group and colour fraction. Tissue samples were taken from fresh material by careful dissection of mantle and foot tissue.

### Sample preparation, melanin oxidation and LC-UV-MS analyses

Two major morphs of *C. nemoralis* (pink banded and yellow banded) were investigated. Their corresponding colour-sorted shell fragments were as follows: pink background; brown band on pink background; yellow background; brown band on yellow background (see Fig. 1A). Three technical replicates of each colour group were performed, with each replicate comprised of up to 8 shells.

Analysis of eumelanin and pheomelanin oxidation products was carried out as previously described^14^ (see main text): In brief, shells were cleaned in deionized water and weighted. Cleaned shell pieces were dissolved in 6 M HCl and centrifuged at 13,000 rpm for 15 min. Residues were washed with HPLC grade water. Samples were treated with proteinase K in 1 M Tris-HCl buffer at 37 °C for 2 h. Treatment was stopped by acidification with 6 M HCl, and samples were centrifuged and washed as described above.

Oxidation reactions and solid-phase extractions were performed as previously described^14^. For each sample, oxidation was performed for 20 h at 25 °C under vigorous shaking using 100 μL H_2_O, 375 μL 1 M K_2_CO_3_ and 25 μL 30% H_2_O_2_. Remaining H_2_O_2_ was decomposed by the addition of 50 μL 10% Na_2_SO_3_ and the mixture was acidified with 140 μL 6 M HCl. Solutions were then centrifuged at 13,000 rpm for 40 min and the supernatants transferred to fresh tubes. Samples were then treated by solid-phase extraction on Phenomenex Strata-X 33 μm polymeric reversed phase columns under vacuum. Columns were first conditioned with 5 mL methanol (MeOH) followed by 5 mL H_2_O. Shell extracts were loaded onto the columns which were then washed with 5 mL 0.3% formic acid. The columns were then dried for 30 min and elution was carried out with 3 mL MeOH followed by 3 mL ethyl acetate. Solvents were removed under constant nitrogen stream at 40 °C and samples were dissolved in 200 μL H_2_O.

Measurements were also performed as previously described^14^. Briefly, a Thermo Fisher Scientific LC-MS system consisting of an Accela HPLC with a Finnigan Surveyor PDA Detector coupled to an LTQ Orbitrap XL mass spectrometer equipped with an electrospray ionisation (ESI) source was employed. Separation was performed on a Phenomenex Gemini C18 column (250 × 2 mm, 5 μm) at 45 °C using a flow rate of 0.2 mL/min. The mobile phase was 0.3% formic acid in H_2_O:MeOH (80:20). UV data were recorded between 200 and 400 nm. Mass spectra were acquired in negative ion mode over an *m/z* range of 120–220.

### Reverse transcription quantitative PCR (qPCR) of melanin pathway genes in *C. nemoralis*

Four genes known to be involved in melanin synthesis and dark pigmentation were chosen for qPCR testing: *Cnem-Tyrosinase* (*Tyr*), *Cnem-Tyrosinase related protein* (*TyrRP*), *Cnem-Yellow-like* (*Yellow*) and *Cnem-Laccase 2* (*Lacc2*). These sequences were identified from within a *C. nemoralis* mantle tissue transcriptome data set using tBLASTx. Primers for qPCR were designed with Primer3 (https://primer3plus.com). Primer sequences and Genbank accession codes are listed in Table 2.

**Table 2:** Primer sequences and accession numbers.

Gene	Forward Primer (5′ to 3′)	Reverse Primer (5′ to 3′)	Amplicon Size (bp)	Genbank accession
*BACT*	CAGAAGCAATGTTCCAGCCA	TGAGCCACCAGACAAGACAA	137	MH035489
*EF1α*	GTACCGGAGAGTTTGAGGCT	GAGTAAGGTGGAGTGGTGCT	133	MH035491
*UBI*	AGAATGCCCCAACAAATGCT	AGAATCAGCCTCTTCTCCGG	121	MH035498
*Tyr*	TCCTACTGGCTTTGGGAGTC	GTATCTTGAAGGGCACTGCG	121	MN590236
*TyrRP*	ACCTCCAACTCCCCTCACTA	CGAGTTCAACATCCGGCATT	125	MN590237
*Yellow*	ACCTCTTCTATGGGGCCTTG	CAACCTCGCTTTCAGTGTCC	117	MN590238
*Lacc2*	CAAGGTCACATCTGGAACGC	TTATCTCTCCTCGTGCGTCC	133	MN590239

The experimental design for RT-qPCRs followed the protocol described in Affenzeller *et al*.^15^: Six sub-adult individuals of *C. nemoralis* were collected at the University of Göttingen. Total RNA from pigmented mantle (producing the band in the shell), unpigmented mantle (producing background coloured shell) and foot tissue was extracted from each individual using Qiazol (Qiagen) according to the manufacturer’s instructions resulting in a total of twelve RNA extractions. These underwent a DNase treatment (RQ1 RNase-free DNase, Promega) according to the manufacturer’s instructions. Nanodrop and agarose gel electrophoresis were employed to verify quality and integrity of RNA. Synthesis of cDNA was carried out with 1 μg of total extracted RNA per sample using Promega M-MLV reverse transcriptase and oligo dTs. Reaction was run at 42 °C for 75 min, followed by 15 min at 70 °C to inactivate reverse transcriptase. The cDNA was stored at −20 °C until further use.

All qPCR runs followed a maximum sample layout, comply with the MIQE guidelines^29^ and included no template controls (NTC) for each primer pair and three inter run calibrators (IRC): elongation factor 1 alpha (*EF1α)*; RNA-directed DNA polymerase (*RNAP);* and Ubiquitin (*UBI)*. Samples were run in triplicate, NTC and IRCs were run in duplicate. Amplification reactions contained 5 μL 2× Rotor-Gene SYBR Green PCR Master Mix, 0.4 μL cDNA, 1 μM final Primer concentration and 4.4 μL ddH_2_O to a final volume of 10 μL. Reactions were run on a Rotor-Gene Q (Qiagen) using Rotor-Gene Q software (version 2.0.2) with the following temperature profile: 5 min initial activation and denaturation at 95 °C; 45 cycles of 5 sec denaturation at 95 °C, 10 sec annealing and extension at 60 °C (data collection at this step); a final melt curve analysis from 60 °C to 95 °C at a rate of 5 sec/1 °C.

### Quantification and statistical analyses

#### LC-UV-MS of melanin oxidation products

Quantitation of melanin oxidation products was carried out by external calibration with standard mixtures (obtained from S. Ito). External calibration was set with 9-point calibration curves. Limit of quantitation (LOQ) was set at 10:1 signal-to-noise ratio. All manual peak integrations of chromatograms and analyses of mass spectra were done in Xcalibur 2.2 Qual Browser (Thermo Scientific, Waltham, USA). Quantitation was based on areas gained from peak integrations of UV chromatograms in a range of 250–290 nm. Each colour fraction was run for three replicates (comprised of up to eight shells each). Statistical analyses (mean and standard deviation calculations) were carried out in Microsoft Excel for Office 365 MSO (16.0.11629.20192).

#### qPCR

Raw fluorescence data were baseline and amplification efficiency corrected in LinRegPCR^30^. Inter run correction was performed using Factor-qPCR^31^. So gained corrected cycle threshold (Cq) values were used to calculate the geometric means of technical replicates. Normalisation and relative expression were calculated based on the Pfaffl method^32^ with *beta-actin* (*BACT*) and EF1α serving as reference genes as previously tested for mantle tissue in *C. nemoralis*^15^.

Descriptive statistical analyses (mean and standard deviation calculations) of six biological replicates for each sample set (pigmented mantle, unpigmented mantle, foot) were carried out in Microsoft Excel for Office 365 MSO (16.0.11629.20192). Statistical comparisons between pigmented mantle and unpigmented mantle, as well as between all mantle samples and foot tissue, were run in PAST 3.15 as t-tests using Mann-Whitney as a significance measure (*p ≤ 0.05, **p ≤ 0.01).

## Supplementary information

Supplementary Information.

Supplementary Information 2.

Supplementary Information 3.

## Data Availability

All data generated or analysed during this study are included in this published article or are available from the Dryad repository (10.5061/dryad.gf1vhhmjs).
