# Supplementary material for: Challenging the concept that eumelanin is the polymorphic brown banded pigment in Cepaea nemoralis
Source: Sci Rep. 2020 Feb 12;10:2442. doi: 10.1038/s41598-020-59185-y (PMC7016172; doi:10.1038/s41598-020-59185-y)
Supplement: Supplementary file 1 — Supplementary Information. [file 41598_2020_59185_MOESM1_ESM.docx]

Phylogenetically relevant Tyrosinase and Tyrosinase-related protein sequences were downloaded from GenBank and aligned in Seaview version 4.7 using the Clustalo algorithm with default parameters. The resulting alignment was filtered for conserved regions using the Gblocks server (<http://molevol.cmima.csic.es/castresana/Gblocks_server.html>) applying the "Allow smaller final blocks", "Allow gap positions within the final blocks" and "Allow less strict flanking positions" options. The following is the G-Blocked alignment used to perform the Bayesian analysis:

#NEXUS

[saved by seaview on Mon Dec 9 10:25:38 2019]

BEGIN DATA;

DIMENSIONS NTAX=27 NCHAR=368;

FORMAT DATATYPE=PROTEIN

GAP=-

;

MATRIX

[1] Cnem_TYRRP_MN590237

HRVLCLVI--AM---------------------------------DLQLPS----L----

-----MGQPQIMDSMRLANILMERPVDGPSIRKECRMLNSTDFQKIVDAINRAKLDTR--

---------VRPNVHDAYSYLHA---------NKGAHGGPAFLPYHRVFIFLYEKLLRIS

LCYWD-----EPE----NMLWSSAWTPELRNWI------------------TNPLERAN-

-DEDVKVVLSK------------------GEISM-PAASVNANVELMHNFVHTYVGGIMG

QVETAAYDPIFWFHHTYIDCLYERFRDQWPAEHGDSAHAPFAPM-RL-GSLRNIDGAQEF

FSKEIVRC

[2] Cnem_TYR_MN590236

PFSVCLAL--GLC---QGKISEIPLPYIGSKFSWSCENIILDS--DKTISYYN-HLLEIA

----MIYLPDNTNGTTVP-RRKRQASRPRCVRREYRAMSDAERERFHSAVNSLKRDTT--

---------VPPNKYDAMANIHA---------LNSAHSGAGFTGWHRLYLRVFEAAVRVC

MPYWD-----DNEL--DEPALSSVWTPAFANWR------------------TAPLIRNS-

-ARAMEDILSR------------------EDITFSPGVQRRFNLELHHNGIHIFCGGTLN

RLSTAAFDPVFFLLHAFVDFIWELFRANPP----TSPHHSSAPT-GF-GSLRQRDAYGIA

ITE-TYEY

[3] Ggor_TYR_Q9BDE0.2

LAVLYCLLFPRACVSSKNLMEKECCPCGQLSGRGSCQNILLSNAPDDRESWPSVFYNRTC

QCSGNFMGFNCGNCKFGFWGPNCTERR-LLVRRNIFDLSAPEKDKFFAYLTLAKHTISSD

YVIGSTPMFNDINIYDLFVWMHYYVSMDALLGIDFAHEAPAFLPWHRLFLLRWEQEIQFT

IPYWDCDICTDEYMGGQHPTNPNLLSPASSSWQIVCSRLEEYNSHQSLCNGTGPLQRNLP

SSADVEFCLSLTQYESGSMDKAANFSFRNEGFASPLTGIADASQSSMHNALHIYMNGTMS

QVQGSANDPIFLLHHAFVDSIFEQWLRRYPEANAPIGHNRESYMVPFIPLYRNGDFFISS

-KDLGYDY

[4] Hsap_TRP1_P17643

LGCIFFPLFPRQCATVEALRSGMCCPCGSSSGRGRCEAVTADSRPDDREVWPLRFFNRTC

HCNGNFSGHNCGTCRPGWRGAACDQRV-LIVRRNLLDLSKEEKNHFVRALDMAKRTTHPL

FVIGNTPQFENISIYNYFVWTHYYSVKKTFLGVDFSHEGPAFLTWHRYHLLRLEKDMQFS

LPYWNCDICTDDLMGSRSNFDSTLISPNSSQWRVVCDSLEDYDTLGTLCNSTGPIRRNLP

EPQDVAQCLEVGLFDTPPFYSNSTNSFRNEGYSDPT-GKYDPAVRSLHNLAHLFLNGTGG

QTHLSPNDPIFVLLHTFTDAVFDEWLRRFPLENAPIGHNRQYNMVPFWPPVTNTEMFVTA

PDNLGYTY

[5] Hsap_TRP2_P40126

L--SCLGCFPRVCMTVDSLVNKECCPCGSQQGRGQCTEVRADTRPDDRELWPRKFFHRTC

KCTGNFAGYNCGDCKFGWTGPNCERKKPPVIRQNIHSLSPQEREQFLGALDLAKKRVHPD

YVIGTQPQFANCSVYDFFVWLHYYSVRDTLLGIDFSHQGPAFVTWHRYHLLCLERDLQFA

LPYWNCDVCTDQLFGAARPDDPTLISRNSSSWETVCDSLDDYNHLVTLCNGTGLLRRNLP

TLKDIRDCLSLQKFDNPPFFQNSTFSFRNEGFDKAD-GTLDSQVMSLHNLVHSFLNGTNA

LPHSAANDPIFVVLHSFTDAIFDEWMKRWPQELAPIGHNRMYNMVPFFPPVTNEELFLTS

-DQLGYSY

[6] Mmus_TRP1_P07147

LAYISLFLFPRECANIEALRRGVCCPCGSSSGRGRCVAVIADSRPDDREAWPLRFFNRTC

QCNDNFSGHNCGTCRPGWRGAACNQKI-LTVRRNLLDLSPEEKSHFVRALDMAKRTTHPQ

FVIGNTPQFENISVYNYFVWTHYYSVKKTFLGVDFSHEGPAFLTWHRYHLLQLERDMQFS

LPYWNCDVCTDDLMGSRSNFDSTLISPNSSQWRVVCESLEEYDTLGTLCNSTGPIRRNLP

EPQDVTQCLEVRVFDTPPFYSNSTDSFRNEGYSAPT-GKYDPAVRSLHNLAHLFLNGTGG

QTHLSPNDPIFVLLHTFTDAVFDEWLRRFPLENAPIGHNRQYNMVPFWPPVTNTEMFVTA

PDNLGYAY

[7] Mmus_TRP2_P29812

L--GCLGCFPRVCMTLDGVLNKECCPCGFLEGRGQCAEVQTDTRPDDREQWPRKFFNRTC

KCTGNFAGYNCGGCKFGWTGPDCNRKKPAILRRNIHSLTAQEREQFLGALDLAKKSIHPD

YVIGTQPQIANCSVYDFFVWLHYYSVRDTLLGIDFSHQGPAFVTWHRYHLLWLERELQFA

LPYWNCDVCTDELLGAARQDDPTLISRNSSTWEIVCDSLDDYNRRVTLCNGTGLLRRNLP

TLKNVQDCLSLQKFDSPPFFQNSTFSFRNEGFDKAD-GTLDSQVMNLHNLAHSFLNGTNA

LPHSAANDPVFVVLHSFTDAIFDEWLKRWPQELAPIGHNRMYNMVPFFPPVTNEELFLTA

-EQLGYNY

[8] Btau_TRP1_Q8WN57.2

LGYMFLVLFPRECATIEALRNGVCCPCGLSSGRGRCEVVIADSRPDDREGWPTRSFNRTC

HCNGNFSGHNCGTCRPGWGGAACDQRV-LTVRRNLLDLSTEEKNRFVRALDMAKRTTHPQ

FVIGNTPQFENISIYNYFVWTHYYSVKKTFLGVDFSHEGPAFLTWHRYHLLQLERDMQFS

LPYWNCDICTDDLMGSRSNFDSTLISPNSSQWRVVCESLEDYDTLGTLCNSTGPIKRNLP

KPQDVAQCLEVGSYDTPPFYSNSTNSFRNEGYSHPT-GRYDPAVRSLHNLAHLFLNGTGG

QTHLSPNDPIFVLLHTFTDAVFDEWLRRYPLENAPIGHNRQYNMVPFWPPVTNIEMFVTA

PDNLGYTY

[9] Ggal_TRP1_O57405.1

LVSLPLLLFPRQCATIESLRSGMCCPCGVSTGRGRCVQVTVDSRPDDREQWPIRFFNQTC

RCNGNFSGYNCGSCRPGWTGPTCSQQI-NIVRRNLLDLSTEERRRFVNALHQAKVTIHPD

IVIGNTPQFENISIYNYFVWSHYYSVRKTFLGVDFSHEGPAFVTWHRYHLLQLERDMQFG

LPYWNCDICSDDLMGARSNFDVSLISQNSSTWRVLCESIEDYDSLGTICNSTGPIRRNLP

EPEDVPQCLEVGIFDTPPFYSNSTDSFRNEGYSDPS-GKYDPAVRSLHNLAHLFLNGTGG

QTHLSPNDPIFVLLHTFTDAVFDEWLRRYPLENAPIGHNREYNMVPFWPPVTNNEMFVTA

PENLGYSY

[10] Amex_TRP1_P55027.2

LPLTAALLFPRQCVTPEALRSGQCCPCGASVGRGRCAPLQVDARPDDREQWPTRFFNNSC

LCAENFSGYDCGSCKPGWVGVNCNQRV-LAVRRNILDLTAQERRRFIAALDLAKRTTHPH

YVIGNSTQFENVSIYNFFVWTHYYSIGKTFLGIDFSHEGPAFVTWHRYHLLQLERDMQFA

LPYWNCDICTDDFMGARSNFDSILLSSNSSQWRVLCESLEGYDTLGTICNSTGPIRRNLP

EPQDVALCLEVGLFDTPPFYSNSSESFRNEGYSEPS-GKYDPSVRSLHNLAHLFLNGTGG

QTHVSPNDPIFVLLHTFTDAVFDEWLRRYPLENAPIGHNRQYNMVPFWPPVSNNEMFVTA

PESLGYSY

[11] Caur_TRP1_P55028.1

CGGMLLLVFPRACVTPEGLRSAQCCPCGALAGPGRCVDVR--MRATTRAL--ARASSRAC

RCNGNFGGFDCGGCAHGFTGDACEQRV-PVVRRNVMQLSADEKRFFVNALDQAKRAPHPD

TVINSTTQFENISIYNLFVWTHYYSVSKTFLGVDFSHEGPGFLTWHRYHLLQLERDMQFA

LPYWDCDICTDELMGARSSSDSSSISSNSSRWRVICESVEEYDTLGTICNSSSPIRRNLP

EPQDVEACLELTAFDSPPFYSTSSDSFRNEGYSAPQ-GNYDPVVRSLHNLAHLFLNGTGG

QTHLSPNDPIFVLLHTFTDAVFDEWLRRYPLENTPIGHNREFNMVPFWPPVTNAEMFVTA

AENLGYSY

[12] Sscr_TRP2_Q4R1H1.1

L--GCLGSFPRVCMTVGSLQAKECCPCGSLEGRGRCAEVQADTRPDDRERWPRKFFDRTC

RCTGNFAGYNCGDCKFGWTGPNCDQKKPLVVRQNIHSLTAQEREQFLGALDLAKNTPHPD

YVIGTQPQIANCSIYDLFVWLHYYSVRDTLLGIDFSHQGPAFVTWHRYHLLWLERALQFA

VPYWNCDVCTDQLLGAARPDDPTLISQNSSSWEIVCDSLDDYNRRVTLCNGTGLLRRNLP

SLKDIEDCLSLKQFDNPPFFQNSTFSFRNEGFDKAD-GTLDSQVMSLHNLVHSFLNGTSA

LPHSAANDPVFVVLHSFTDAIFDEWMKRWPQELAPIGHNRMYNMVPFFPPVTNEELFLTA

-DQLGYSY

[13] Btau_TRP2_Q95119.2

L--GCLGCFPRVCMTVGSLQAKECCPCGSREGRGQCAEVQTDTRPDDRERWPRKFFDRTC

RCTGNFAGYNCGNCRFGWTGPKCDQKKPLVVRRDVHSLTPQEREQFLDALDLAKYTLHPD

YVIGTRPQIANCSIYDFFVWLHYYSVRDTLLGIDFSHQGPAFVTWHRYHLLWLERDLQFA

LPYWNCDVCTDQLLGAARQDDPTLISQNSSSWEIVCDSLNDYNRRVTLCNGTGLLKRNLP

TLKDIQNCLSLKKFDSPPFFQNSTLSFRNEGFGKAD-GTLDSQVMNFHNLVHSFLNGTSA

LPHSAANDPVFVVLHSFTDAIFDEWMKRWPRELAPIGHNRMYNMVPFFPPVTNEELFLTA

-DQLGYSY

[14] Ggal_TRP2_O93505.1

VGLSYLSCFPRVCMTVEAIRSKRCCPCGVLQGRGWCQGVQVDTQPDDRERWPLKFFNQSC

WCTGNFAGYNCGDCKFGWTGPDCSVRKPPVVRKNIHSLTVEEREQFLDVLDRAKTTIHPD

YVIGEEPQIANCSIYNYFVWLHYYSVRDTLLGIDFSHQGPAFVTWHRYHLLLLERDLQFA

LPYWDCDVCTDQLFGAPRPDDPGLISLNSSRWQIVCNSLDDYNRLVTLCNGSGLLQRRLP

TAEDVRRCLSRHEFDSPPFFRNSSFSFRNEGFNKPE-GALNSPMLNLHNLAHSFLNGTRV

LPHAAANDPIFVVLHSFTDAIFDEWMKRWPEELAPIGHNRLYNMVPFFPPVTNDQLFQTA

-EQLGYTY

[15] Hsap_TYR_P14679

LAVLYCLLFPRACVSSKNLMEKECCPCGQLSGRGSCQNILLSNAPDDRESWPSVFYNRTC

QCSGNFMGFNCGNCKFGFWGPNCTERR-LLVRRNIFDLSAPEKDKFFAYLTLAKHTISSD

YVIGSTPMFNDINIYDLFVWMHYYVSMDALLGIDFAHEAPAFLPWHRLFLLRWEQEIQFT

IPYWDCDICTDEYMGGQHPTNPNLLSPASSSWQIVCSRLEEYNSHQSLCNGTGPLRRNLP

SSADVEFCLSLTQYESGSMDKAANFSFRNEGFASPLTGIADASQSSMHNALHIYMNGTMS

QVQGSANDPIFLLHHAFVDSIFEQWLRRYPEANAPIGHNRESYMVPFIPLYRNGDFFISS

-KDLGYDY

[16] Mmus_TYR_P11344

LAVLYCLLFPRACASSKNLLAKECCPCGQLSGRGSCQDILLSSAPDDRESWPSVFYNRTC

QCSGNFMGFNCGNCKFGFGGPNCTEKR-VLIRRNIFDLSVSEKNKFFSYLTLAKHTISSV

YVIGSTPMFNDINIYDLFVWMHYYVSRDTLLGIDFAHEAPGFLPWHRLFLLLWEQEIRFT

VPYWDCDICTDEYLGGRHPENPNLLSPASSSWQIICSRSEEYNSHQVLCDGTGPLLRNLP

SSADVEFCLSLTQYESGSMDRTANFSFRNEGFASPLTGIADPSQSSMHNALHIFMNGTMS

QVQGSANDPIFLLHHAFVDSIFEQWLRRYPEANAPIGHNRDSYMVPFIPLYRNGDFFITS

-KDLGYDY

[17] Btau_TYR_Q8MIU0.2

LAALYCLLFPRACASSKSLTEKECCPCGRLSGRGSCQDVILSTAPDDRESWPSIFYNRTC

QCFSNFMGFNCGSCKFGFRGPRCTERR-LLVRRNIFDLSVPEKNKFLAYLTLAKHTTSPD

YVIGTTPLFNDVSVYDLFVWMHYYVSRDTLLGIDFAHEAPGFLPWHRLFLLLWEQEIQFT

IPYWDCDVCTDEYMGGRNPANPNLLSPASSSWQIVCSRLEEYNSRQALCNGTGPLLRNLP

SSADVEFCLSLTQYESGSMDKAANFSFRNEGFADPVTGIADASQSSMHNALHIYMNGTMS

QVPGSANDPIFLLHHAFVDSIFEQWLRKYPEANAPIGHNRESYMVPFIPLYRNGDFFISS

-KDXGYDY

[18] Ggal_TYR_P55024.1

LFAMGLLLFPRVCANTQSLLRKECCPCGERSNRGTCQRILLSQAPDDREDWPSVFYNRTC

RCRGNFMGFNCGECKFGFSGQNCTERR-LRTRRNIFQLTISEKDKFLAYLNLAKNIPSKD

YVIGSNPMFRNINVYDLFVWMHYYASRDTLLGIDFAHEAPGFLPWHRAFLLLWEREIQFT

IPYWDCVICTDEYMGGQHPTNPNLLSPASSSWQVICTQSEEYNSQQALCNATGPILRNLP

SSSEVEFCLTLTQYESGSMDKMANYSFRNEGFADPHTAISNISQSGLHNALHIYMNGSMS

QVQGSANDPIFILHHAFVDSIFERWLRRYPAANAPIGHNRENYMVPFIPLYRNGEFFISS

-RELGYDY

[19] Cgig_TRP1_XP_011416067.1

------------------------MP-----------AIFPISRLSRFVQWMN-GL----

-----FYLPRG---------------NELRVRKEYRLLSDEERRSYHQAILLLKNDRT--

---------VLPNKFDAIASLHH---------ASGAHGGPGFLGWHRIYLTLFENALRVT

IPYWD-----DAEL--PDPRRSIMWSPLFRRFT------------------TGPLRRDS-

-KTDLENVFSR------------------WEISN-PSAEDRYNLELLHNHVHVWIGEQMS

RIESSSYDPAFFSHHAFVDCLWEEFRQRYPRIVGDQGHQPLVSM-GL-GRLLVVDGINDI

FTQRIYSC

[20] Cgig_TRP3_XP_011428737.1

-MILCFLL--GTQ---T-VGQGVFIN--PLDFDIHDESMFATS--EADVTWLN-SL----

-----FSLPEE---------------GETRERKEYRLLTEEEREYFHRAVNMLKNDTT--

---------VSLNKYDLLANIHS---------NTAAHGGPGFLGWHRVFLLLFENALRAT

LPYWD-----DQPL--SHPSESVIWSDLFRGWN------------------TGLLHRQS-

-VHDLRNILEE------------------GNISY-PDTKSSKNLEQLHNNVHVWVGGLMR

KIEIGAFDPVFYVLHTFIDKVWEDFRVHYPEFYGRRNHASFAPM-GL-GNLVVIDGISDV

WNKNVVY-

[21] Pmax_TYRB2_1_AHZ34291.1

PYGLTLILGIKY----------DPIPNYNSTFNNDCAEFISNSFLDDQINFIK-SLDREA

----MSLLYGSERTKRQ---------TRSPVRRECRTLSQNDWGRLSHAIRRLK---F--

---------DPGNEYDTMAHTHT---------IDNSHDGSNILGWHRLFLFLFEIALRVV

LCYWD-----DYLLRGRGQVQSAAFSHELANFP------------------TGPLRRNLV

RPHIVDMIERD-----PNIRSHGQL-VDGTGFTD-PLSGERTSLEAEHNNAHVAVGALMA

IIPNAAYDPLFFFHHCYIDYVWELFRRKYLGHGGP-AHARNAPMRGLIPGWRNIHGYSNF

FSRRYRYA

[22] Pmax_TYR_AHZ34297.1

------------------------------------------------------------

---------------------------------MCALSAEEWRDLAAAIVALKRNT-A--

---------VAPNLFDSLAAVHT---------IGNAHFGPNFLGWHRIYLYYYEIALRVA

LCYWD-----DFSM--DSPERSVMFSSEYRDWI------------------LRQPEQRVY

DG--VREILTD-----PQLIRTANVSVGG-------IAPIQRTIEGLHNNVHVWVGQIMS

GVDTAPQDPVFYFHHCYIDYFWERYREKYPRDMGGNAHLPGAPMAVF-RWVNNERGFMNA

FTRYLFRY

[23] Pmax_TRP_P86952.1

LLGKVFL-GVGFC-----MLMQDPKRQGNEPASPDCLKAFMAYAEKEQINYLW-SLERET

----QSLLHNHRRRKRQA--------VYLPVRKECRLLSELERQNLFYTVRSLKMD-T--

---------SNPNEYDTLANLHR---------QPHAHDGSNFLGWHRVYLMYYERALRVT

LCFWD-----EFNLGMDNWEYTAVFSSDFRDWP------------------LGLTESDPF

DSRAASSIFYN-----PNTIIHSTITWEGDTITNSQGQTRNITIEGEHNNVHNWVGGAMG

FLDPAPQDPIFFFHHCYIDYVWERFREKYPGHGNETLHDANYPMIGF-EWYRNIDGYSDY

FTQNVYRY

[24] Pmar_TRP2_H2A0L1.1

LFGKVIL-GVGFC-----MLMQDPKRQGNEPASPDCVKAFMAYAEKEQINYLW-SLERET

----QSLFHNHRRRKRQA--------VFLPVRKECRLLSEFERQNLFYTIRSLKMD-T--

---------SNPNEYDTLANLHR---------QPHAHDGSNFLGWHRVYLMYYERALRVT

LCFWD-----DFNLGMDNWEYTAVFSSDFRDWP------------------LGLTESDPF

DSRAASSIFYN-----PNTIIHSTVTWEGDTITNSQGQTRNITIEGEHNNVHNWVGGAME

FLDPAPQDPVFFFHHCYIDYVWERFREKYPGHGNETLHDANYPMIGF-EWFRNIDGYSDY

FIQNVYRY

[25] Pmar_TRP1_H2A0L0.1

TLQSLIV-YGALC-----MLQTENVKTDSKYLDPKCVTIFMDQYREDQMNYIF-SLERAM

----MRKHHINDKRHKRQ--------AMTRPRQECRTLTDDARNNLFNTIVDLKAP-S--

---------NGMSRYDTIAGLHR---------IANAHMGANFLGWHRLYLDMFEMALQVV

LCYWD-----DFLMPGTSQVNTVSFSAELRFWR------------------LGRTLQRLT

RPGVVDLIATD-----PRINTNSQIVFRGPDP---DTGRPGHSWEDEHNNTHVWVGGVMQ

NVVSSPQDPVFWFHHTYVDYVWELFRQKYPADASG-PHAPDAPMIGF-DMLQNRDGYSDE

HSRM--YA

[26] Hcum_TYR_APC92581.1

IFLF--FL--GCVTSTNAYLEFLPMPTVGHSLNRHCVNSYYFRSEDFDMHYLK-FLKR-R

----IFD--NHFRAKRQS-DINPSPPSGFRVRKEYRRLTDSERTAYHSVLNVMKRN----

------------GEYDTFARIHS---------LGQFHDGPNFLGWHRIYLAYFEEAVRLS

LPFWD-----DFPL--SDPTQSVLWSATFSGWV------------------VSPLIRNS-

-KQDVDTVLTR------------------SEITF-PRETGTYNLEIYHNRVHNWVGGNME

LLDTAAFDPAFFLHHAFVDYVWELFRLRYPNVVGDHAPQRE-MH-AF-PQHRASDGYANY

WTDSWYTY

[27] Hcum_TRP1_APC92582.1

-MSFCSFLMISLLPYVRATVEQTNVPTPSNAIQSWCTNVYKWQHDRELVHYLS-DQV---

----LQSVHHIGRV-----KRQVVSSGPKRRRKEIRMLTERELDLYFRAVRAAKANTT--

---------TAPNVYEALAEFHT---------SISAHGGCNFFGWHRVYLLMYENMLRVT

IPYWD-----EARM--EQPTSTVLFTNRFSGWQ------------------TGPLIRNT-

-DEAIVNVTRM------------------SEICG-ADSAIESDLEFHHNGIHRWVDGQMA

MLQTSPLDPAFWNHHTFVDYVWEAFRINYPANPTAELHAPDAAL-GF-AEMTVIDGLSNT

FTAEIYEY

;

END;

The following parameters were implemented in MrBayes version 3.2.6 to perform the phylogenetic analysis.

begin mrbayes;

log start filename=mrbayes.log;

set autoclose=yes nowarn=yes;

execute Tyrosinase_related_renamed_ALIGNED_GBlocked_faa.nxs;

lset rates=gamma;

prset aamodelpr=mixed;

mcmcp nruns=2 ngen=50000000 printfreq=1000 samplefreq=1000 nchains=8 savebrlens=yes temp=0.2 stoprule=yes;

mcmc;

sump;

sumt;

quit;

**Bayesian phylogenetic analysis of Tyrosinase and Tyrosinase-related proteins.** The tree has been mid-point rooted and node labels are the posterior probabilities of 50 million generations. Accession numbers for all sequences are provided in square brackets. TRP1, Tyrosinase Related Protein 1; TRP2, Tyrosinase Related Protein 2.
