# Supplementary material for: Challenging the concept that eumelanin is the polymorphic brown banded pigment in Cepaea nemoralis
Source: Sci Rep. 2020 Feb 12;10:2442. doi: 10.1038/s41598-020-59185-y (PMC7016172; doi:10.1038/s41598-020-59185-y)
Supplement: Supplementary file 2 — Supplementary Information 2. [file 41598_2020_59185_MOESM2_ESM.docx]

Phylogenetically relevant Laccase sequences were downloaded from GenBank and aligned in Seaview version 4.7 using the Clustalo algorithm with default parameters. The resulting alignment was filtered for conserved regions using the Gblocks server (<http://molevol.cmima.csic.es/castresana/Gblocks_server.html>) applying the "Allow smaller final blocks", "Allow gap positions within the final blocks" and "Allow less strict flanking positions" options. The following is the G-Blocked alignment used to perform the Bayesian analysis:

#NEXUS

[saved by seaview on Mon Dec 9 16:15:49 2019]

BEGIN DATA;

DIMENSIONS NTAX=25 NCHAR=305;

FORMAT DATATYPE=PROTEIN

GAP=-

;

MATRIX

[1] Cnem_MN590239_LACC

VAADGGLRGIITANPGPSIHVCENDIIRVRVHNKMTTIHWHGVLQHGSPYMDGVSMVTQC

PIPTYTAFTYVFGTHFWHSHAGLQRADGLFGHLVVRQAEPHWGVYDFDLPEHELLVNDWW

HPSTILINGKGMDTPRELFTVKKGFRYRFRIISISVDEHSLEIIASDGAPVSPMEVESFN

IIAGERYDFVLTANQDNYLIRVAILHYEGAEEGQEVAMDFMNGITNLFPPAPPLHPTHLH

GHGFRVLNLDKPPLKDTVGGYTVVRFHANNPGIWFLHCHIEYHVEIGMGLIFQVGPPRFP

TCGSW

[2] Csat_AO_P14133.1

WSPDCVENIVMGINPGPTIRANAGDIVVVELTNKLVVIHWHGILQRGTPWADGTASISQC

AINPGETFTYRFGTYFYHGHLGMQRSAGLYGSLIVDPPGRS-EPFHYDEE-INLLLSDWW

HPQSILINGKGQFCAPFILHVQPKKTYRIRIASFAIGNHELLVVEADGNYVQPFVTSDID

IYSGESYSVLITTDQENYWVSITLLNYLPNSASKLL-LNTINNVSLALPPTPYLHPWHLH

GHDFWVLNLKNPPLRNTVYGWTAIRFVADNPGVWAFHCHIEPHLHMGMGVVFAEGPPKAL

ACGST

[3] Tver_LAC4_Q12719.1

VSPDGFTRAAVLANPGPLITGNKGDNFQINVIDNLTSIHWHGFFQKGTNWADGAAFVNQC

PIATGNSFLYDFGTFWYHSHLSTQYCDGLRGPMVVYDPDPHADLYDVDDETTIITLSDWY

HSDSTLINGLGRFTDLAVITVEQGKRYRMRLLSFSIDGHNMTIIEADAVNHEPLTVDSIQ

IYAGQRYSFVLTADQDNYFIRAAILRYSGASEVDPLNLDFINDVSF-VSPTVPVHPFHLH

GHTFSIVNFVNPVRRDVVGDNVTIRFTTDNPGPWFLHCHIDFHLEAGFAIVFSED--TTT

PSTAW

[4] Post_LAC2_Q12739.1

VSPDGFARSAVVARPGVLVQGNKGDNFQLNVVNQLTSIHWHGFFQAGSSWADGPAFVTQC

PVASGDSFLYNFGTFWYHSHLSTQYCDGLRGPFVVYDPDPHLSLYDIDNADTVITLEDWY

HPDSTLINGKGRYSPLAIINVESNKRYRFRLVSFSIDGHSLLVIEADAVNIVPITVDSIQ

IFAGQRYSFVLTANQDNYWIRAAILRYAGATEDDPINLAMINGVPF-LPPTAPVHPFHLH

GHTFDVINFDTPARRDVVNDNVTIRFVTDNPGPWFLHCHIDWHLEIGLAVVFAED--ISA

PPAAW

[5] Tvil_LAC4_Q99055.1

VSPDGFTRAAVLANPGPLITGNKGDNFQINVIDNLTSIHWHGFFQKGTNWADGAAFVNQC

PIATGNSFLYDFGTFWYHSHLSTQYCDGLRGPMVVYDPDPHADLYDVDDETTIITLSDWY

HSDSTLINGLGRFTDLAVITVEQGKRYRMRLLSFSIDGHNMTIIEADAVNHEPLTVDSIQ

IYAGQRYSFVLTADQDNYFIRAAILRYSGASEVDPLNLDFINDVSF-VSPTVPVHPFHLH

GHTFSIVNFVNPVRRDVVGDNVTIRFTTDNPGPWFLHCHIDFHLEAGFAIVFSED--TTT

PSTAW

[6] Prad_LAC_Q01679.2

VSPDGFSRQAVLAEPGPLIAGNKGDNFQINVIDELTTIHWHGFFQHGTNWADGPAFINQC

PIASGDSFLYNFGTFWYHSHLSTQYCDGLRGPFVVYDPDPYLDQYDVDDDSTVITLADWY

H-DTTLINGLGRCSDLAVISVTKGKRYRFRLVSFSIDGHSLNVIEVDATNHQPLTVDELT

IYAGQRYSFILTADQDNYWIRAAILRYDGADVVEPLNFNLINGVTF-VPPTVPVHPFHLH

GHTFSVVNYVNPVQRDVVGDNVTIRFDTNNPGPWFLHCHIDWHLEAGFAVVFAED--INP

VPQDW

[7] Cmel_LAC_P37064.1

WAPNCNENIVMGINPGPTIRANAGDSVVVELTNKLVVIHWHGILQRGTPWADGTASISQC

AINPGETFFYNFGTFFYHGHLGMQRSAGLYGSLIVDPPGKK-EPFHYDGE-INLLLSDWW

HPQTILLNGRGQFCAPYIFHVSPKKTYRIRIASFAIGNHQLLVVEADGNYVQPFYTSDID

IYSGESYSVLITTDQENYWVSVTLLNYLPNSVSKLL-LNTINDVSLALPPTPYLHPWHLH

GHDFWVLNLKNPPLRNTVYGWTAIRFVADNPGVWAFHCHIEPHLHMGMGVVFAEGPTKAL

ACGGT

[8] Cmax_LAC_P24792.2

WAPDCNENIVMGINPGPTIRANAGDTVVVELINKLVVIHWHGILQRGTPWADGTASISQC

AINPGETFFYNFGTFFYHGHLGMQRSAGLYGSLIVDPPGKK-EPFHYDGE-INLLLSDWW

HPQTILLNGRGQFCAPYIFHVMPKKTYRIRIASFAIGNHPLLVVEADGNYVQPFYTSDID

IYSGESYSVLITTDQENYWVSVTLLNYLPNSVSKLL-LNTINDVSLALPPTPYLHPWHLH

GHDFWVLNLKNPPLRNTVYGWTAIRFVADNPGVWAFHCHIEPHLHMGMGVVFAEGPTKAL

ACGGT

[9] Tvil_LAC5_Q99056.2

VTPDGITRAAVLAGPGPLITGNKGDEFQINVIDNLTTIHWHGIFQAGTNWADGAAFVNQC

PIATGNSFLYDFGTFWYHSHLSTQYCDGLRGPLVVYDPDPNASLYDVDDDTTVITLADWY

HPDSVLINGLGRFTNLTVITVTQGKRYRFRLVSFSIDGHNMTIIEVDGVNHEALDVDSIQ

IFAGQRYSFILNANQDNYWIRAAILRYDTAEDIEPMSLDFINNETF-VPPTVPVHPFHLH

GHTFSVVNYANPVRRDTVGDNVTIRFTTDNPGPWFLHCHIDFHLEAGFAIVWGED--ANP

VPTAW

[10] Post_LAC1_Q12729.1

VSPDGFTRSAVVARTGVLVQGNKGDNFQLNVLNQLTSIHWHGFFQSGSTWADGPAFVNQC

PIASGNSFLYDFGTFWYHSHLSTQYCDGLRGPFIVYDPDPHLSLYDVDNADTIITLEDWY

HADSTLINGKGRFSALAVINVESNKRYRFRLISFSIDGHSLQVIEADAVNIVPIVVDSIQ

IFAGQRYSFVLNANQDNYWIRAAILRYAGATEDDPINLAMINGSPF-KAPTAPVHPFHLH

GHTFDVINFDTPARRDVVNDNVTIRFVTDNPGPWFLHCHIDWHLEIGLAVVFAED--ITA

PPAAW

[11] Abis_LAC1_Q12541.1

LAPDGFERDTVVINPGTLIQVNKGDSVRIPLHNKLVSIHWHGFFQARTSGQDGPSFVNQC

PQPPNTTFTYEFGTFWYHSHLSTQYCDGLRGAFIVYDPDPLRHLYDVDDESTVITLAEWY

HQDSGLINGKGRFTPFAVVNVRRGKRYRLRVIAFSVDNHSLVFMEADGVEHDPVEVQNVD

IYAAQRVSVILHANQDNYWIRAAILRYHGARHVEPITLNIINGISY-ISPTVPVHPFHLH

GHNFDVVNFKNPPRRDVYGGNTTFRFFTDNPGAWFLHCHIDWHLEAGLAIVFAEAPQSQI

TPQDW

[12] Tver_LAC5_Q12717.1

VTPDGITRAAVLAGPGPLITGNKGDEFQINVIDNLTTIHWHGIFQAGTNWADGAAFVNQC

PIATGNSFLYDFGTFWYHSHLSTQYCDGLRGPLVVYDPDANASLYDVDDDTTVITLADWY

HPDSVLINGLGRFTNLTVITVTQGKRYRFRLVSFSIDGHNMTIIEVGGVNHEALDVDSIQ

IFAGQRYSFILNANQDNYWIRAAILRYDTAEEIEPMSLDFINNETF-VPPTVPVHPFHLH

GHTFSVVNYANPVRRDTVGDNVTIRFTTDNPGPWFLHCHIDFHLDAGFAIVFAED--ANP

VPTAW

[13] Bbas_LACOpS5_J5JH35.1

MAPDGYEQHVLAINPGPLIEANWGDEVVIHVTNNMTAIHWHGIRQLNNNAHDGVPGVTQC

PIPPGGSYTYRWGTSWYHSHFSLQYSVGLQGPMIIHGPTAN---Y--DEDLGTVMLQDWS

HLSNSLINGKNIFGERSEWHFEKGKRYRMRLVNFAIDGHNLTVIANDFVPIEPYTTDNVI

ISMGQRYDVIVEANADNYWLRAGIIRYDPQSTALPNLYRHLNDTYLWIDWSKPTHPMHLH

GHDFFLLQLKNPPRRDTARGYMVFAYKTDNPGAWLIHCHIAWHSSQGLGMQMLER--TED

EDQAL

[14] Tcuc_LAC2_Q02075.1

VAPDGVTRNAVLVNPGPLITANKGDTLKITVRNKLTTIHWHGLLQHRTAEEDGPAFVTQC

PIPPQESYTYTMGTYWYHSHLSSQYVDGLRGPIVIYDPDPYRNYYDVDDERTVFTLADWY

HPDSGTINGKGKYENLYTLKVKRGKRYRLRIINFGVQGHKCTIIEADGVLTKPIEVDAFD

ILAGQRYSCILKADQDSYWINAAAALVAGEAELDKLDFGLINNVSY-SPPDVPTHPLHLH

GHAFDVVNYVNPPRRDVVDAGVRIQFRTDNPGPWFLHCHIDWHLEEGFAMVFAEASQSVK

PDGQW

[15] Abis_LAC2_Q12542.1

LAPDGFERDTVVINPGTLVQVNKGDSVRIPVNNKLVSIHWHGFFQARTSGQDGPAFVNQC

PQPPNTTFTYEFGTFWYHSHLSTQYCDGLRGAFVVYDPDPLGHLYDVDDETTVITLAEWY

HQDSGLINGKGRFTPFAVVNVEQGKRYRFRVIAFSVDNHNLTFMEADSVEHDPVEIQNVD

IYAAQRVSVILNANQDNYWMRAAILRYKGAPEVEPITLNIINGISY-ISPTVPVHPFHLH

GHNFDVVNFVNPPRRDVYGGNTTFRFFTDNPGAWFLHCHIDWHLEAGLAIVFAEAPQSQI

TPQDW

[16] Tcas_LAC2_NP_001034487.2

VLADGVERGILTANPGPSIQVCEGDKVVIDVENHIVTLHWHGVWQRGSQYYDGVPFVTQC

PIQQGNTFRYQWGTHFWHAHTGLQKMDGLYGSVVIRQPDPNSHLYDYDLTTHVMLLSDWM

HPESLLINGKGQFTPLEVFTITPGRRYRFRMINLTIQGHDLTLIATDGEPVHPVRVNTII

SFSGERYDFVINADQGAYWIQLGILRYAKGPYQPSLPFRFIDEISYMAPPAPLIHPFHLH

GYAFNVIQFNLPPAKDTINGYVVLRLRANNPGFWLFHCHFLFHIVIGMNLVLQVGPPNFP

TCGDH

[17] Tcas_LAC2B_AAX84203.2

VLADGVERGILTANPGPSIQVCEGGKVVIDVENHIVTLHWHGVWQRGSQYYDGVPFVTQC

PIQQGNTFRYQWGTHFWHAHTGLQKMDGLYGSVVIRQPDPNSHLYDYDLTTHVMLLSDWM

HPESLLINGKGQFTPLEVFTITPGRRYRFRMINLTIQGHDLTLIATDGEPVHPVRVNTII

SFSGERYDFVINADQGAYWIQLGILRYAKGPYQPSLPFRFIDEISFTFPPSPPLHPFHLH

GYAFNVVQFNLPPSKDTINGYVIFRFRADNPGYWLFHCHFLFHIVIGMNLIIHVGPHNFP

RCGNH

[18] Tcas_LAC1_AAX84206.1

IPGDGNKRSIIVVNPGPSVEVCLGDEVIIDVVNHLTTIHWHGHHQKNSPYMDGVPFVTQC

PIHPGMTFRYHFGTHFWHSHSGFQRSDGTFGPFIVRVPNPHAKLYDYDLSSHVITILDWT

KPDTILVNGFGRFVPTARFTVEQGYRYRFRVINVSIDNHTLSVISTDGSDFNATEVDSLV

TYAGERFDFIVTADQDVYWMHFAVLEYKGTQTNYPVSYDFLNYISMKLQSFPLLHPFHLH

GHSFRVVNLVDAPLKDTVGGFTIIRFKATNPGYWLFHCHIEFHVEVGMALVFKIGPKDFP

QCGDY

[19] Bgla_LAC15_XP_013074978.1

VPADGGLRGILTVNPGPSIQVCENDVIRVRVHNKMTSIHWHGLLQRGTPYMDGVSMVTQC

PIPAHSSFTYVFGTHFWHAHSGLQRADGLYGHLVIRQAEVHHDLYDFDLPEHVLVVNDWW

HPSSVLINGKGAFTPREMFNVTLGKRYRFRVASVSVDNHSLEIIASDGQPVQPLTVDAFN

IFAGERYDFILTANQGNYLMRFAILHYEDVEMTEDVAMDFMNDISNMMPPAPPLHPTHLH

GHGFRIVNIHKAPIKDTVGGYTIVRFHANNPGIWFLHCHVEYHVEIGMGLLFQVGPRRFP

TCGNW

[20] Bgla_LAC2_XP_013074984.1

--------------PGPSIQVCENDVIRVRVHNKMTSIHWHGLLQRGTPYMDGVSMVTQC

PIPAHSSFTYVFGTHFWHAHSGLQRADGLYGHLVIRQAEVHHDLYDFDLPEHVLVVNDWW

HPSSVLINGKGAFTPREMFNVTLGKRYRFRVASVSVDNHSLEIIASDGQPVQPLTVDAFN

IFAGERYDFILTANQGNYLMRFAILHYEDVEMTEDVAMDFMNDISNMMPPAPPLHPTHLH

GHGFRIVNIHKAPIKDTVGGYTIVRFHANNPGIWFLHCHVEYHVEIGMGLLFQVGPRRFP

TCGNW

[21] Acal_LAC1_XP_012944262.1

VPADGVSRGIRTANPGPGIQVCEGDTVEVTVDNRMTSIHWHGILQNGTGHMDGVAMVTQC

PVTSHSKFVYRFGTHFWHAHAGFQRSDGLFGSLVVRRPEPHLHLYDYDLPEHVLIVNDWM

FPDSMLINGQGRYTPRAEFKVKPNHKYRFRVISVSVDDHTMMIIASDGNSFQPFVADSFN

IFAGERYDFVLYADKRNYWIRVAILRYEGAPLEDPLAMDFINHITSVLPSSPPLHPMHLH

GYNFRVVTTSRAPLKDTVGGYTIIRFRADNPGVWFFHCHIEFHSEIGMGLLFQVGPKNFP

RCGNW

[22] Bgla_LAC4_XP_013067388.1

VSADGVMRGIKTANPGPRVEVCEGDTIEVTVMNAMTSIHWHGIHQRGTPFMDGVAMLTQC

PIPAHAKFTYRFGTHFWHAHAGVQRADGLFGSLVIRRPEPQYLLYDYDLTDHVIIVNDWL

APELMLINGRGQYTPRATFTVKPGKRYRFRIINFSVDSHTMTMIASDGHSFAPYTVDMFN

IFSGERYDFVLYADKRNYWIRVAILKYEGAPDDLPLAMDFINHISTLLPPVPPLHPMHLH

GYSFRVITKTKAPLKDSVGGYTIVRFKADNPGIWFFHCHIEFHAEIGMGLFFQVGPKNFP

KCGDF

[23] Cvir_LAC2_XP_022312912.1

VAADGTRRGIITVNPGPGIHICYGDTVVVNVINKLVSIHWHGLHQRNTPHMDGVSMLTQC

AIHAHSSFQYKFGTYFWHSHSGTQRMDGLFGPLVIRQEEPHLGFYDHDLPEHAILINDWL

VPASMLINGKGTFTPHEVLTVEHGKRYRIRLISFSVDNHSLTIIATDGFPVKPMTVESLN

VFAGERFDIVIAATQGSYWIRTAILRYQGSNNSLPMSMDFINYISNLLPPAPPLHPMHLH

GYSYRVVNLNSSVIKDTVGGYTIVRIHATNPGFWFFHCHIDFHAEIGMGLILQVGPHKFP

MCGNW

[24] Myes_LAC1_XP_021368004.1

IPADGNSRAVFVINPGPGIHVCEGDTVIVNVHNKMMSIHWHGILQHNSPHMDGVGMLTQC

PIHSFSSFQYRFGTHFWHGHSGLHRSDGIFGAFVVRQAEFHHGLYDYDLPEHTMLVNDWL

MPASMLINGRGAHTPHEVFNVERGRRYRFRIISLSIDNHTLRVISSDGNPFNHIDVETIN

IFAGERYDVVILADQGNYWVRAAILRYNGSAEELPLAMDFINYISNILPPSPPLHPMHLH

GHKFRVINLTSPVDKDTVGGYVILRIHADNPGFWFMHCHIEFHAAIGMGFVLQVGPENFP

RCGNW

[25] Pcan_LAC1_XP_025077846.1

VPADGTSRGILTANPGPGIQVCHGDEIIVNVYNKLTSIHWHGLLQRGSQYMDGTSMVTQC

PITTHSTFQYRFGTHFWHSHAGLQRADGLFGSLIVRQPDIHSPLYDFDLPEHVIIVTDWL

NAESMLINGKGAFTPYADFTVRPTFRYRFRVISFSIDNHTLIVIASDGGDFDPIIVDSFN

IFSGERFDFILIADKKNYWVRAAVLRYTGAPPGLPLAMDFINHISFSSTAVPPLHPMHLH

GYKFRVVKLSRAPLKDTVGGYTIIRFKADNPGIWLFHCHIAFHGEIGMALLIQAGPKNFP

TCNNW

;

END;

The following parameters were implemented in MrBayes version 3.2.6 to perform the phylogenetic analysis.

begin mrbayes;

log start filename=mrbayes.log;

set autoclose=yes nowarn=yes;

execute Tyrosinase_related_renamed_ALIGNED_GBlocked_faa.nxs;

lset rates=gamma;

prset aamodelpr=mixed;

mcmcp nruns=2 ngen=50000000 printfreq=1000 samplefreq=1000 nchains=8 savebrlens=yes temp=0.2 stoprule=yes;

mcmc;

sump;

sumt;

quit;

**Bayesian phylogenetic analysis of Laccase proteins.** The tree has been mid-point rooted and node labels are the posterior probabilities of 50 million generations. Accession numbers for all sequences are provided in square brackets. .
