# Supplementary material for: Challenging the concept that eumelanin is the polymorphic brown banded pigment in Cepaea nemoralis
Source: Sci Rep. 2020 Feb 12;10:2442. doi: 10.1038/s41598-020-59185-y (PMC7016172; doi:10.1038/s41598-020-59185-y)
Supplement: Supplementary file 3 — Supplementary Information 3. [file 41598_2020_59185_MOESM3_ESM.docx]

Phylogenetically relevant Yellow sequences were downloaded from GenBank and aligned in Seaview version 4.7 using the Clustalo algorithm with default parameters. The resulting alignment was filtered for conserved regions using the Gblocks server (<http://molevol.cmima.csic.es/castresana/Gblocks_server.html>) applying the "Allow smaller final blocks", "Allow gap positions within the final blocks" and "Allow less strict flanking positions" options. The following is the G-Blocked alignment used to perform the Bayesian analysis:

#NEXUS

[saved by seaview on Tue Dec 10 10:16:14 2019]

BEGIN DATA;

DIMENSIONS NTAX=39 NCHAR=220;

FORMAT DATATYPE=PROTEIN

GAP=-

;

MATRIX

[1] Cnem_YEL_MN590238

GKVLYEWLDVEYDWYEADGRYDRTHSFISGVYLTVPRGVPSTLNRISVLTPFPDWCNALQ

CAMSMEVDSGLMYVIDTGRSCPPKLVVYDLADLNDIVIDRGGPGCKTARWIYITDAVDSK

LVVFNLVTNKTHIDYEPGDGSNITVNGVSYPIDGLAISSEYVYYCALGSKKLYQVPSSVV

DNLFYGALIKNSVYKWVVRDDERLFSACNAEKFRAGACPC

[2] Amel_MRJP2_O77061.1

LNVIHEWKYFDYDFAIQSGEYDHTKNYPFDVFVTILRGVPSTLNVIRLLKPYPDWCSKIV

SAFKIAIDFDRLWVLDSGLVCAPKLHVFDLKTLVSLAVQAIDL---ANTLVYMADHKGDA

LIVYQNADDSFHRFDYDPRYAKMTIDGESFGICGMALSPNNLYYSPLASHGLYYVNTAPF

GVLFVGLVGNSAVGCWVAQNDRTLVLSNRMQKILIRNTHC

[3] Dmel_YEL_P09957.1

LQERYSWSQLDFAFALASGDYIPQNALPVGVFVTVPRGIPATLTYIPELIPYPDWANSIT

TAYRIKVDCGRLWVLDTGTVCPYAVNVFDLTTIANIAVDI-GK-NCDDAYAYFADELGYG

LIAYSWELNKSWRFFPDPLRGDFNVAGINFGIFGMSLSPRTLYFSPLASHRQFAVSTRIL

GIELFNLIDQNAVGCWVDRDDVGLVLSDRMPVFLIENTVC

[4] Dsim_YEL_P62407.1

LQERYSWNQLDFAFALASGDYIPQNALPVGVFVTVPRGIPATLTYIPELIPYPDWANSIT

TAYRIKVDCGRLWVLDTGTVCPYAVNVFDLTTIANIAVDI-GK-NCDDAYAYFADELGYG

LIAYSWELNKSWRFFPDPLRGDFNVAGINFGIFGMSLSPRTLYFSPLASHRQFAVSTRIL

GIELFNLIDQNAVGCWVDRDDVGLVLSDRMPVFLIENTVC

[5] Dyak_YEL_Q9BI17.1

LQERYSWNQLDFAFALASGDYIPQNGLPVGVFVTVPRGIPATLTYIPELIPYPDWANSIT

TAYRIKVDCGRLWVLDTGTVCPYAVNVFDLTTIANIAVDI-GK-NCDDAYAYFADELGYG

LIAYSWEQDKSWRFFPDPLRGDFNVAGINFGIFGMSLSPRTLYFSPLASHRQFAVSTRIL

GIELFNLIDQNAVGCWVDRDDVGLVLSDRMPVFLIDNTVC

[6] Dere_YEL_Q9BI23.1

LQERYSWNQLDFAFALASGDYIPQNALPVGVFVTVPRGIPATLTYIPELIPYPDWANSIT

TAYRIKVDCGRLWVLDTGTVCPYAVNVYDLTTIANIAVDI-GK-NCDDAYAYFADELGYG

LIAYSWEQDKSWRFFPDPLRGDFNVAGINFGIFGMSLSPRTLYFSPLASHRQFAVSTRIL

GIELFNLIDQNAVGCWVDRDDVGLVLSDRMPVFLIENTVC

[7] Dpse_YEL_Q9BI18.2

LQERYSWNQLDFAFAMASGDYIPQNALPVGVFVTVPRGIPATLTYIPELIPYPDWANSIT

TAYRIKVDCGRLWVLDTGTVCPYAVNVFDLTTIANIAVDI-GK-NCDDAYAYFADELGYG

LISYSWELNKSWRFFPDPLRGDFNVAGINFGIFGMSLTPRTLYFSPLASHRQFAVSTRIL

GVELFNLIDQNAVGCWVDRDDVGLVLSDRMPVFLIENTVC

[8] Dgau_YEL_Q9GP81.1

LQERYSWNQLDFAFALASGDYIPTNALPVGVFVTVPRGIPATLTYIPELIPYPDWSNSIT

TAYRIKVDCGRLWVLDTGTVCPYAINIFDLTTIANIAVDI-GK-NCDDAFAYFADELGYG

LISYSWELNKSWRFFPDPLRGDFNVAGINFGIFGMSLTPRTLYFSPLASHRQFAVSTRIL

GVELFNLIDQNAVGCWVDRDDVGLVLSDRMPVFLIENTVC

[9] Dsub_YEL_O02437.1

LQERYSWNQLDFAFALASGDYIPTNALPVGVFVTVPRGIPATLTYIPELIPYPDWANSIT

TAYRIKVDCGRLWVLDTGTVCPYAINIFDLATIANIAVDI-GK-SCDDAFAYFADELGYG

LISYSWELNKSWRFFPDPLRGDFNVAGINFGIFGMSLTPRTLYFSPLASHRQFAVSTRIL

GVELFNLIDQNAVGCWVDRDDVGLVLSDRMPVFLIENTVC

[10] Dmad_YEL_Q9GP71.1

LQERYSWNQLDFAFALASGDYIPTNALPVGVFVTVPRGIPATLTYIPELIPYPDWANSIT

TAYRIKVDCGRLWVLDTGTVCPYAINIFDLTTIANIAVDI-GK-NCDDAFAYFADELGYG

LISYSWELNKSWRFFPDPLRGDFNVAGINFGIFGMSLTPRTLYFSPLASHRQFAVSTRIL

GVELFNLIDQNAVGCWVDRDDVGLVLSDRMPVFLIENTVC

[11] Dmel_YELf2_Q9VG08.2

MIEVFRWKQMDFYNDDPNASYIPYNNVPMGAFVTMPRGIPSTLNYIPKLRAYPNFAENLV

SVYRTSVDCQRLWFIDTGMLRRPSIWVVDLATLASITVDV-KAGQCGDAYAYIPDLVYRR

LYVYHLRNDRIWSFNFDPLSGDLSIGGQTFGIFSITLGARDAYFHPMASTNEFVVSNRVL

GVIFFDEIQRNGVGCWVDSNAEDMVMSNSMPIFAKRGTVC

[12] Dmel_YELf_Q9VG09.1

MIEVFKWKQLDFYNSSSSGSFIQYNNVPQGVFVTVPRGIPSTLNYIPHLRAYPNLEQNLV

SVYRTSVDCGRLWFVDTGMLRHPSIWVIDLANLASITIDV-GARRCNDAYAYIPDLVNRR

LHVYHLRSDRIWSFNFDPLSDNLNIGGQTFGIFSATLGSRDVFFHPMASTNEFVVSNRVL

GVIFFAEVQKSGVGCWVYSNSSEMVMSNSMPIFAKRGTVC

[13] Amel_MRJP1_O18330.1

LPILHEWKFFDYDFAILSGEYDYKNNYPSDIFVTMLRGVPSSLNVIPLLQPYPDWCSGIV

SASKLAIDCDRLWVLDSGLVCSPKLLTFDLTTLSSLAVQSLDCNTNSDTMVYIADEKGEG

LIVYHNSDDSFHRFDYDPKFTKMTIDGESYGISGMALSPNNLYYSPVASTSLYYVNTEQF

GVLFFGLVGDSALGCWVAQSDETLVLSNKMQKMLILNTRC

[14] Amel_MRJP5_O97432.1

MNVIHEWKYLDYDFAMQSGEYDHTKNYPFDVFVTVPRGVPSSLNVIRLLQPYPDWCSGIV

SAYKIAIDFDRLWILDSGIICSPKLHVFDLNTLVSLVVQAMDP---VNTIVYMADDKGDA

LIVYQNSDESFHRFDYDPKYIKMMDAGESFGIFGMALSPNNLYYSPLSSRSLYYVNTKPF

GVLFFGLMNNSAIGCWVAQNEETLALSMKLQKFLIMNTRC

[15] Amel_MRJP4_Q17061.1

LNVIHKWKYLDYDFAIQSGEYDRTKNYPLDVFLAVIRGVPSSLNVVRLLQPYPDWCSGIV

SAHKIAIDYERLWVLDSGLVCSPKLFAFDLNTLVSLTVQAMDS---TNTMVYMVDNK-NT

LIIYQNADDSFHRLNHNSD--KMSDQQENLKVYGMALSPHNLYYNSPSSENLYYVNTESL

GVLLFGLANN-TLSCWVARNEDTLALSDRNQNVLIRNSRC

[16] Amel_RJP57_1_Z26318.1

MKVIYEWKHIDFDFAIKSGEFDHTKNYPFDVFVTIERGVPSSLNVVPLLRPYPDWCSGIV

SAFKIAVDFDRLWVLDSGLVCSPKLLTFDLKTLVSLAVQAIDR---TNTMVYIADEKGEG

LIMYQNSDDSFHRFDYDPRYTKLTVAGESFGIYGIALSPNNLYYSPLLSHGLYYVDTEQF

GVLFLGLVGNSGIACVVAQNEETLALSNRMQKILMRNTRC

[17] Amel_MRJP6_AY313893.1

MNVIHEWKYIDYDFAIQSGEYDYTKNYPFDVFLAVIRGVPSSLNVICLLQPYPDWCSGIV

SAYKIAIDFDRLWVLDSGLICSPKLLAFDLNTPVSLVVQAMDP---MNTTVYIADDRGDA

LIIYQNSDDSFHRFDNDLRYSELAVAGESFGIFGMALSPNNLYYSPLTSHSLYYVNMEPF

GVLFFGLVNNSAIGCWVAQNEKTLALSNRMQKILIKNTRC

[18] Amel_MRJP8_AY398690.1

LKVIYEWKYIDYDFAIQSGDYNYTMNYLLDTFVIIMKGVPSSLNVIPLLAPYPDWCSGIT

SAYKIEIDCDRLWVLDSGLICPPQLLVFDLNTLVTLSVQLLSCEVNGSTLVYIGDNEGFA

LIIYNNSDNSFQRFASDPRYTTFTINGESFGIFGMALSPQNLYYSALSSHNLNYVNTEQF

GVLFFGLVGDTSLACWVAKNKETLAVSNRIQKVLIKNTRC

[19] Amel_MRJP7_BK001420.1

LKVMHEWKYIDYDFAIQSDEYDHTKNYPFDVFVTVLRGVPSSLNVIRLLQPYPDWCSGIV

SAYSIAIDFDRLWVLDSGLVCFPKLLVFDLNSLKSLAVQAISS---VNTLVYIADNKGDG

LIVYQNSDDSFHRFNYDPRYTKMTVEGESFGIYGMALSPNNLYYSPLASRDLYYVNTKPF

GILFFGLVNNTAVGCWVAQNEETLVLTNRMQKILLENTRC

[20] Amel_YELF_like_AY661557.1

IKTIYSWNVIEYNFLISNGDYIEENNMPNGMFITIPRGVPSNLNFFPKLNPYPNWIDSII

NIIRVRVDCDRLWGVDTGVDHQPRIIIIDLKTFVDLVIDV-DPNNCDNTYAYISDLSGYA

LVVYSWAKNDSWRFYFDPRYGNYNINGFNFGLFGLSLSAKILYFHAMSSIAEFSVSTEVL

GVDYFTQINRNGIACWVAENNTTMVLSDNFQQLACKKKDD

[21] Amel_MRJP9_DQ000307.1

FQVKYQWKYFDYNFAIQSGEYNYKNNVPIDVFVTILRGVPSSLNVIPLLEPYPNWCSGIT

SVYRIAIDWDRLWVLDNGISCPSQIVVFDLKNVVTPIVQSFD---YNNTWVYIADVEGYA

LIIYNNADDSFQRFVYDPRYTKYTINDESFGILGMALSHQNLYYSAMSSHNLNYVNTKQF

GALFFGLVSDTALGCWVAKNNDTLIVSNKYQKILIRYTRC

[22] Dmel_YEL_E3_NP_650288.1

LHTLHQWTNLSLGD--------GNRFLPVDVFLTIPRATPFTLATVPRLEPYPNECSGIT

SAIRTYIDCWRLWVVDSGQVCPPQILTFDLVKLVVDLAERGTPNRCVGGRAYIADAWGYG

LIVFDSLTGRSWRMKPSP----LLRLGRSSGIFTVSLSPRFLYFHTLNSFNEMRVPLSLI

GNLYCSLISLGALVKWVAYNPHKIALSSQPKLFLLANTPC

[23] Amel_MRJP1_XP_396824.1

LPQSLIFSGLSLDWYETSGRYIARNVIATRAILALPRGVPFTLGILPKVAPFPCWCQALQ

SAVDIVLDQDILWVLDVGIVCPPKVVGVDAKTLQYMAVDYAEDG---QVYVYISDAGTGA

IIVYNVTTDTGYRV-----AGCTDKP----DALYIALVRPVLYFTFLGSNRMFAIKAVNL

ATIFFRIKGDSSIYMWLVQKAGDCVIESNFQDY-------

[24] Amel_YEL_XP_396825.2

LPETIKWTGGNFEWYKSNGKYISKNVIATRIIVALPRGIPATLAKIATLIPYPCWCTALQ

NVVDLYLDQNILWILDTGVVCPAKVLAIDVTTLQYVVSDYTQDG---RVFIYVSDAASRA

ILVYDVTSGRGYRV-----SMGCTRR----DVLYLALLRTCLIFTYLSSSRMFSIRTEHL

SALFFRYEGEADVYRWKVYTSAECVLESNFPDY-------

[25] Bmor_YEL8_ABC96701.1

YERTHYWRLFGYDIEEHEKFLIQKNLVPNHVIVSIPRGIPFTINKMPLLMPYPTSSENII

SVYKTVEDCERYWFVDTGFI-------------RSLTIDYIFP--CNETFAYISDDNGDA

VIAFSFEEKRFWRS--E--AWTFPIPQS---ILRYA--EKSPNET----FVHY---SDIL

GVLYDRNYDGN-----------------------------

[26] Amel_MRJP1_XP_001122824.1

LEIVFQWKYLDWLWNQTLG---NAFTQDVDIVTSPQWGVPISLSLVPLLVPYPDWCDSII

SVYRLAIDCNRLWVVDTGRVCPTKILIFDLATLVTPIVDVG--KMCSDTYLYVADVDQNG

LLIYDFYHDYSWRFGPDDDATNITIAGESFGTLGMSLSPRYLYFNSLASYRQKFTDTYSL

GVIFFQLVQLTAIACWIAQDEETLFNTNRLQKTIIKGTNC

[27] Bmor_YEL_fa_NP_001037424.1

LTEVFAWKQLTYNINETGRFFVQYNNVPMGVFITVPRGIPSTLNYVPALRPYPSLGSSLV

SVYRTRADCGRLWMVDTGRLQPPAIVVFDLNTLASITIDT-KS-GCDTAHAYVPDLTTYG

IIVYSLRDNDSWRFHFNPIAGTLNIAGQSFGIFSLTIKKDTAYFHPLISTHEFAVSTCLL

NVMFYAEVGRDAFSCWLAKDAIRLLMANSLPRFAIYGTTC

[28] Bmor_YEL_fb_NP_001037428.1

MKQLYAWKQLGYDFGDSEKFFIQYNNVPTGIFLTVPRGIPSTLNYIPLLKPYPNPVSSLT

SVYRTAIDCARLWMVDTGLLKPPAILAYNGLTLTSLTIDV-EPTACGEAHAYITDLATNG

LIVFSLGARAFWRFVHDEKALNFTVAGNVIGLFSIALSDRLAYYHPMVSTHEYAIDTAVL

SVLFYANVARDGIVCWIVQDREKLVLVNKMPVFLIKGTVC

[29] Bmor_YEL_b_NP_001037430.1

FRVVYEWNAIDFEWYLNTSQYIPQNVLISGIFLTMPRGVPATLATIPKLKPFPSWCNALQ

FVQNIEIDNGIMWILDNGRVCPPSIVLIDLKSLNDLVVDNRDGD---YAYITDNSAVDPG

IIVFRRSDKRSWKMRAANDATFFRINGTTVNIDGIALGPRKIYYSPLASFHLYAINASVL

GNLYYGLLGIASIAEWIARDPNYIVIINRLYNSEDVTPVM

[30] Bmor_YEL_f2_NP_001037432.1

FKYERSWKLFSYDIEDHEKFLIRQNLIPYNFIISIPRGIPFTVNYILVLRPFPNAGKELI

SVFTMVEACSRIWFVDTGYLKPAALLLFNKYELRSLSVDFILP--CSESYVYITDDTTRD

LIVFSLQDLRFTKA--D--SWKFMVPRI---CREMSTAQKNPYLH----TRTL----DVL

----DRV---------------------------------

[31] Amel_YEL_h_NP_001091687.1

MELVYAWSTIDYTYAIFDGDFITENNLPLGLFITLPKGIPVTLTTVPKLRPYPNWCDGLT

SVFRIQVDCDRLWILDSGKVCPPAIFIFDLTTYTNIVVDI-RNEDCGSAIAYVSDVFRYG

LLIYDFFKDSSFRFYPDPLASKYELHGLKFGIFGMALSPRTLFFHPMSSFREFAVSTSIL

GVMFFNMVTRDSVWCWIGTSNLSLVLSNKLAMYAVKDTXC

[32] Dmel_YEL_e2_NP_650289.2

TPIVFEWKNLQYGFVLRNGRYNPDSPIPIDIFVTSPRGVPFSLGYVSEIQAYPSYCDGLT

SVYRVHIDCGQMWVLDSGEICAPQVMVFDLATIFADIRDPPPSGQCKDVFAYLADPTSKA

IVVYDVVGQSSWRTYPDAKFGTHTVAGESFGPLALATTPRHLIFHALSNELELAIPLDIL

GFLFCGFLEPIGIFGWLAINPATLLLSDRLQKILLQGRGC

[33] Tcas_YEL2_NP_001161784.1

FVPAFSWNYINFSW---EFGYVPGEDVIAGIYLALPRSSRVTLASIPLLSPYPNWCDAIQ

NVLSMEIDDGIMWVLDARRVCPPKIILLDLNDLNDIVVD---GD---FAYVSDTTSSDPG

IFVYNRKLKTSWKMFGDPAAVDFTAQGVQNHVNGIALCCRAFFYMPQTSLHIFSTSNSVL

DNIYYGILPLNAVGKW-EQNDEIILITNSISKF-------

[34] Tcas_YEL3_NP_001161785.1

FQLEYQWHYVNYTWAVIKRKYVPENVAITGIYLAMPQGVPVTLGKIPLVHPYPSWCTTLQ

SVQNMEIDSGVMWVLDGFRICPPKLVLFDLNNLKDIVIDSTNDG---FAYITDNSFLDPG

LIVYSRGQNRAWKMFDEEDASGFVVGNLTFPLDGIALSPRLLFYSPLAGMNLYAIKSKVL

GNLYYTLPPLYGVGKW-MAEDKNMLLTNNINKY-------

[35] Tcas_YEL4_NP_001161786.1

FEITHQWSYINFTWAIKSRQYIPEHVAMTGIYIALPKGVPVTLAYIPLLTPYPDWCEGVK

AVQSFEVDNGVLWVLDGFRLCPTKLICYDLKTFNDIVVDD--SG---YVYITDASFIDPG

LIVFSVRENRAWKMFPQMGAANFVVDGMVFTINGIALTPRVLLYCSLVGFSVFGISTETL

GNLYYGLLPLYGVGKW-YQNRSTMVLADSIHKF-------

[36] Tcas_YEL5_NP_001165862.1

FEITHQWSYINFTWAIDSGDYIPENVAMTGIYIALPKGVPVTLAYFPLLTPYPDWCEGIK

AVQSMEIDSGVMWVLDGFRVCPTKLIWYDLNTLNDIVVDD--TG---YAYITDNSAIDPG

LIVFSVRENRAWKMFPQIEAANFVVDDWPFPIDGIALTPRVLLYCSLTGFSVFGISTEVL

GNLYYGLLPLYAVGKW-YQNRSTVVLANTINKF-------

[37] Tcas_YELf_NP_001161780.1

LHEEFSWTRINYVWPDTNPDYVYENNIPMGAFVTVPRGVPSTVNFVVPLIPYPDWGYRFV

SVYRVAVDCDRLWFVDTGLIQPTALVLMDLKTLASLTIDI-TNNNCRDAFAYIPDVGGYG

LVVYSLRQNKAWRFYLENTVGEFSIGGHEFGIFSIAITDRDAYFHSMAGVNLYKVSTRIL

GVLFLGLVNQNALGCWVQRSDEKMVLTNTMPVLAIRGTKC

[38] Tcas_YEL1_NP_001161783.1

FYVIRQWKYLNFTWATATGDYIPENNIVSGIYLTLPRGVPATLARIPPLEPFPSWCNNLQ

NVQNVEIDKGQVWIIDGGRVCGAKLVVYGLRELYDLVVDDTDGG---FAYITDNSAIDPG

IIVFSVKENRAWKMTADPSARHFTANGVPINIAGIALGPREVYFSPLSSLHLYSINTSSL

GILYYTLLANNAIGRWIAQDSKYLVLMNRLNQFDNFNYDM

[39] Tcas_YELe_NP_001161779.1

LEVVNQWNLLDFQF----NNFRPERNVFTGLFLAVPRGVSATLAVIPMLQAYPNWCDGLT

SVYRIRADCNRLWVLDSGVNCPPKLVVFDLETLTNLVIDETGHGGCERAFVYMSDTAAPG

LVVYDSRHDTAWRMFPDPDFSDYTVAGERFGVVGLTHSPGILYFQPLATDRLFSIPTSAL

DTIFFSPVRETSIAAWIAYDPQALLLSTRFQKFRVAS---

;

END;

The following parameters were implemented in MrBayes version 3.2.6 to perform the phylogenetic analysis.

begin mrbayes;

log start filename=mrbayes.log;

set autoclose=yes nowarn=yes;

execute Tyrosinase_related_renamed_ALIGNED_GBlocked_faa.nxs;

lset rates=gamma;

prset aamodelpr=mixed;

mcmcp nruns=2 ngen=50000000 printfreq=1000 samplefreq=1000 nchains=8 savebrlens=yes temp=0.2 stoprule=yes;

mcmc;

sump;

sumt;

quit;

**Bayesian phylogenetic analysis of Yellow proteins.** The tree has been mid-point rooted and node labels are the posterior probabilities of 50 million generations. Accession numbers for all sequences are provided in square brackets. .
